# Biofilm Formation by *Staphylococcus epidermidis* and Its Inhibition Using Carvacrol, 2-Aminobenzemidazole, and 3-Indole Acetonitrile

**DOI:** 10.1021/acsomega.2c05893

**Published:** 2022-12-21

**Authors:** Muhammad Umair Akbar, Asma Haque, Sadia Liaquat, Peter Schierack, Aamir Ali

**Affiliations:** † Department of Bioinformatics and Biotechnology, Government College University, Faisalabad, Faisalabad 38000, Pakistan; ‡ Institute of Biotechnology, Brandenburg University of Technology, Cottbus−Senftenberg, Universitätsplatz 1, Senftenberg D-01968, Germany; § National Institute for Biotechnology and Genetic Engineering College, Pakistan Institute of Engineering and Applied Sciences (NIBGE-C, PIEAS), Jhang Road, Faisalabad 38000, Pakistan

## Abstract

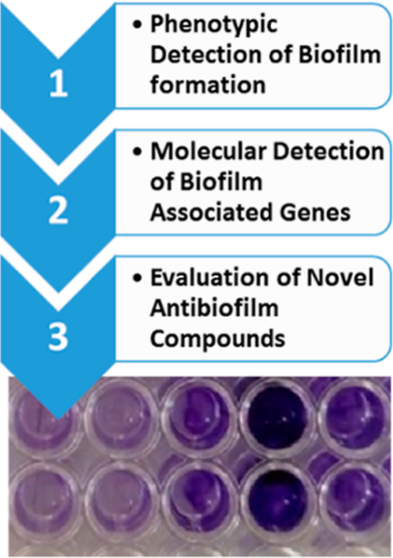

Biofilm-associated bacterial infections are problematic for physicians due to high antimicrobial resistance in biofilm-forming bacteria. *Staphylococcus* species, particularly *Staphylococcus epidermidis,* cause severe infections particularly associated with clinical implants. In this study, we have detected the biofilm formation potential of clinical *S. epidermidis* isolates using phenotypic and genotypic approaches in nutrient-rich and nutrient-deficient growth conditions. The Congo red agar method determined the biofilm formation potential with limited efficacy. However, the tissue culture plate method adroitly classified the isolates as strong, moderate, weak, and non-biofilm producers with five (10%) of the isolates as strong biofilm producers. Ten biofilm-associated genes were targeted, and the *fruA* gene was found to be the most prevalent (20%). Three antibiofilm compounds, carvacrol, 2-aminobenzemidazole, and 3-indole acetonitrile, were assessed against strong biofilm-producing *S. epidermidis* isolates. To the best of our knowledge, this is the first report of genotypic and phenotypic detection of biofilms formed by clinical *S. epidermidis* isolates from this region. The use of 3-indole acetonitrile against these biofilms and toluene as a solvent is novel. The study highlights the significance of biofilm and antibiofilm potential of the studied compounds for effective treatment and control of *S. epidermidis* infections.

## 1 Introduction

*Staphylococci* cause moderate to severe clinical infections particularly on skin and other body parts. These infections are frequently related with catheters and other persistent implanted biomedical devices.^1^ Although the utilization of ingrained clinical gadgets is crucial for the wellbeing of the persistently sick patients, the bacterial colonization on these embedded materials can cause significant adverse effects.^2^ Different species of the bacterial genus *Staphylococcus*, particularly *Staphylococcus aureus,* are well-known to colonize human mucosal layers or tissues, causing a wide range of skin diseases,^3^ whereas *Staphylococcus epidermidis* has been reported as being predominant among the bacterial species colonizing the implanted devices with its major virulence factor of framing biofilms on various polymeric surfaces.^4^ A biofilm is composed of multifaceted bacterial cell groups embedded in a network of an extracellular polysaccharide matrix that enables adherence of these microbes to the target surfaces.^5^ Factors causing biofilm formation include articulation of polysaccharide intracellular adhesin (PIA), which facilitates cell to cell adhesion and is the result of the icaADBC gene family.^6^ The presence of the icaADBC gene family has been reported in *S. epidermidis* isolated from medical devices.^7,8^ However, there are some other proteins involved in biofilm formation of *S. epidermidis* such as cell wall anchored protein, accumulation-associated protein (Aap), extracellular matrix binding protein (Embp), and surface protein C (SesC).^9^

Biofilms shield the residing bacteria from the host defense systems, antimicrobials, and other environmental stresses and, thus, provide resistance to anti-infection treatments.^5^ Various techniques are accessible to detect biofilm formation by *S. epidermidis* including the tube method,^10^ Congo red agar (CRA) method,^11,12^ tissue culture plate (TCP) method,^13^ bioluminescence test, and light or fluorescence microscopic methods.^14^ The biofilm formation by certain pathogenic bacteria contributes toward their multiple drug resistance,^15^ and thus, the treatment and management particularly of implant infections become difficult. Therefore, non-antimicrobial compounds having adequate antibiofilm potential are being investigated. Some reports have shown the antibiofilm activity of certain compounds such as mefenamic acid, acetaminophen, acetylsalicylic acid,^16^ and some nonsteroidal anti-inflammatory drugs against certain bacteria.^17,18^ The search for new molecules preventing biofilm formation and/or dispersing mature biofilms is ongoing. The current study was aimed to explore the biofilm formation potential of *S. epidermidis* isolates from Pakistan, where no comprehensive data are available regarding the infections associated with *S. epidermidis*(19,20) and to evaluate some naturally occurring and synthetic compounds for their antibiofilm activity against these isolates.

## 2 Materials and Methods

### 2.1 Bacterial Isolates

A total number of 50 *S. epidermidis* isolates were taken from institutional stock cultures. These bacteria were originally isolated from various clinical specimens and infected devices from hospitals in different regions of Punjab and Islamabad, Pakistan. The cultures were revived in tryptic soya broth (TSB) and streaked on nutrient agar plates for the analysis of colony morphology. Molecular confirmation was achieved by the polymerase chain reaction (PCR) targeting the *gseA* gene as reported earlier.^21^ Briefly, the 1 mL overnight TSB cultures were centrifuged, and the cell pellet was washed once with sterile distilled water and then suspended in 300 μL of distilled water. The cell suspension was kept at 100 °C in a heating block for 10 min, immediately transferred to ice for 15 min, and then centrifuged at 10,000*g* for 5 min. The supernatant cell lysate was stored at −20 °C till further use as a template in the PCR. In addition to the template, each of the 25 μL PCR volumes contained PCR buffer, 2.5 mM MgCl_2_, a 0.7 M concentration each of dNTPs, a 0.1 μM concentration each of the two primers, and 1 U of *Taq* polymerase. The thermal cycler conditions were kept the same as those reported earlier.^21^ The amplified products were electrophoresed on 1.5% agarose gel by setting the voltage at 90 V and photographed under UV illumination. The confirmed isolates were subjected to biofilm formation assays.

### 2.2 Biofilm Formation Assay

All the isolates were screened for their ability to form biofilms by the TCP method as described earlier^22^ with minor modifications in the media composition and the duration of incubation. Two media were used to evaluate the biofilm formation by *S. epidermidis* isolates: TSB supplemented with 1% glucose (as an enriched medium) and M9 (as a nutrient-deficient medium).^23^ Each of the isolate was grown in TSB at 37 °C for 18 h with 180 rpm shaking [optical densities (ODs) of all the cultures were checked, determined to be approximately 10^8^ CFU/mL] and diluted as 1:100 in fresh TSB (supplemented with 1% glucose) and fresh sterile M9 medium. A 200 μL volume of diluted cultures was coated in each well of the round-bottom polystyrene 96-well plates (SPL cat # 31396). A *S. aureus* isolate was used as a positive biofilm-forming control, whereas 200 μL of both sterile media were used as negative controls. The TCPs were placed at 37 °C for 48 h for biofilm formation. After the incubation, the entire contents of the plates were removed by inversion and gentle tapping. Each of the wells was washed with 200 μL of normal saline to remove the planktonic bacteria and media. The plates were stained with 0.1% (w/v) crystal violet at a concentration of 200 μL per well and kept at room temperature for 15 min. The stain was removed by inversion and gentle tapping followed by washing of the plates once with normal saline. The de-staining was performed using a 200 μL per well concentration of 30% glacial acetic acid for 15 min at room temperature. The well contents were aspirated and transferred to a new plate keeping the well-pattern intact. The OD of the new plate was measured at 630 nm using an ELISA reader (Diamate Bio Technologies Ltd. UK). Each of the isolates and controls were added in triplicate wells, and the experiment was performed twice. The average OD_630_ values of the particular sterile medium were used as a standard for classification of the biofilm-forming isolates according to Stepanović.^24^ The cutoff OD (OD_C_) was calculated as three standard deviations above the mean OD of the negative control (OD_C_ = OD_Neg_ + 3 × SD_Neg_). Each of the isolate was designated as the non-biofilm former (OD_630_ ≤ OD_C_) or weak (OD_C_ < OD_630_ ≤ 2 × OD_C_), moderate (2 × OD_C_ < OD_630_ ≤ 4 × OD_C_) or strong biofilm former (4 × OD_C_ < OD_630_). Additionally, the OD values of each isolate in both media were also statistically compared by the unpaired *T* test with Welch’s correction using GraphPad Prism software version 7.0.

### 2.3 Biofilm Detection on CRA Plates

For phenotypic detection of biofilm formation on agar plates, the CRA method was used as previously described.^25^ The medium contained 37 g/L brain heart infusion broth (cat # CM1135, Oxoid, UK), 10 g/L bacteriological agar (cat # LP0011, Oxoid, UK), 5 g/L sucrose, and 0.8 g/L Congo red dye (Winlab, UK). The Congo red solution and the remaining media components were prepared and autoclaved separately and allowed to cool till ∼55 °C. The Congo red solution and media were mixed and poured into the plates. A 3 μL volume containing overnight culture of each isolate was spotted on the agar plates and incubated at 37 °C for 48 h. The isolates were classified according to the previous reports^1^ as strong (black colonies with rough consistency), weak (partial or complete black colonies with smooth consistency), and non-biofilm formers (pink moist colonies).

### 2.4 Molecular Detection of Biofilm-Associated Genes

Different biofilm-forming genes reported earlier to be contributing in biofilm formation^7,8^ were targeted during this study. The primer sequences, product sizes, and names of targeted genes are given in Table 1.

**Table 1:** Primers Used for *S. epidermidis* Identification and to Target Biofilm-Associated Genes

gene	associated functions	primers (5′ to 3′)	amplicon size (bp)
*gseA*	serine protease	ATCAAAAAGTTGGCGAACCTTTTCA	124
		CAAAAGAGCGTGGAGAAAAGTATCA	
*atlE*	a major autolysin	AACGAAGCAAGTAGCACC	108
		ACACCACGATTAGCAGAC	
*fruA*	fructose specific permease	GTGCAGGTTGCATGTCTA	179
		AAGTGACCCTGTATCGTTTA	
*sarA*	a global regulator	ATTTGCTTCTGTGATACGGT	103
		TGAACACGATGAAAGAACTG	
*sigB*	a σ factor	TACTCTAAGGGACAATCACATC	119
		GGTACTAAGAAGGCTTCAAACT	
*icaA*	PIA production	AGTTTCAGGCACTAACATCC	295
		CGCAGTTACAGGTAATCCAC	
*icaB*	PIA production	ATG GCT TAA AGC ACA CGA CGC	526
		TAT CGG CAT CTG GTG TGA CAG	
*icaC*	PIA production	ATA AAC TTG AAT TAG TGT ATT	989
		ATA TAT AAA ACT CTC TTA ACA	
*icaD*	PIA production	AGG CAA TAT CCA ACG GTA A	371
		GTC ACG ACC TTT CTT ATA TT	

### 2.5 Antibiofilm Assay

Three antibiofilm compounds: one natural [carvacrol (CAR), Sigma cat # 282197] and two synthetic [2-aminobenzemidazole (2-AB), Sigma cat # 171778 and 3-indole acetonitrile (3-IA), Sigma cat # 129453] were tested against three strong biofilm-producing *S. epidermidis* isolates (in each media): isolates MU-5, MU-6, and MU-45 in TSB medium and isolates MU-8, MU-18, and MU-45 in M9 medium. Each of the compounds was tested at three different concentrations. For the antibiofilm assay, a fresh colony of each isolate was inoculated in TSB and incubated at 37 °C for 18 h with 180 rpm shaking (containing 10^8^ CFU/mL). The growth was diluted as 1:100 in the respective media (supplemented TSB or M9), and a 200 μL volume of diluted cultures containing antibiofilm compounds was coated in each well of the 96-well plates. For CAR, 1, 2, and 3 mM (in ethanol) final concentrations were tested;^26^ for 2-AB, 50, 100, and 200 μg/mL (in toluene)^27^ final concentrations, whereas for 3-IA, 0.25, 0.5, and 1 mg/mL (in toluene) final concentrations were used.^28^ Bacterial cultures in respective media without any antibiofilm compound were used as positive controls, cultures containing solvents only (ethanol and toluene) were used as “solvent controls” (to rule out any effect of the used solvent on biofilm formation), whereas sterile media were used as negative controls. The plates were kept at 37 °C for 48 h, and the formed biofilms were detected as described earlier and compared with the solvent controls to determine the antibiofilm effect of the tested compounds. Each sample was tested in triplicate wells, and the experiment was performed twice.

## 3 Results

A total of 50 isolates were identified as *S. epidermidis* on the basis of their colony morphology on nutrient agar as white, 2–3 mm in diameter, raised colonies with a round shape with complete edges. All the isolates were confirmed as *S. epidermidis* by successful amplification of the 124 base pair (bp) fragment of the *gseA* gene using the PCR.

### 3.1 Phenotypic Detection of Biofilm Formation

**Figure 1:**
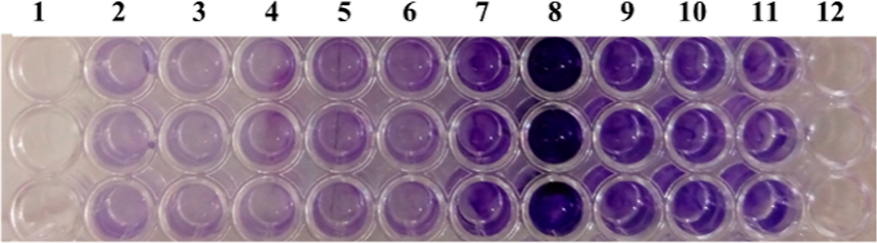
Classification of biofilm-producing *S. epidermidis* isolates using the TCP method. Lane 2: negative control (without inoculation), lane 3: non-biofilm producer, lanes 4–6: weak biofilm producers, lanes 7 and 9–11: moderate biofilm producers, lane 8: strong biofilm producers, and lane 1 and 12 were kept empty.

The confirmed 50 isolates were allowed to form biofilms in TSB media (supplemented with 1% glucose), and the TCP method detected three (6%) isolates (MU-5, MU-6, and MU-45) as strong biofilm formers, six (12%) as moderate biofilm formers, and 36 (72%) as weak biofilm formers, while 5 (10%) isolates were detected to be non-biofilm formers according to Stepanović’s classification.^24^ In the case of M9 medium, three (6%) isolates (MU-8, MU-18, and the same MU-45) were found to be strong biofilm formers, with 11 (22%) as moderate biofilm formers, 23 (46%) as weak biofilm formers, and 13 (26%) as non-biofilm formers (Figure 1).

**Figure 2:**
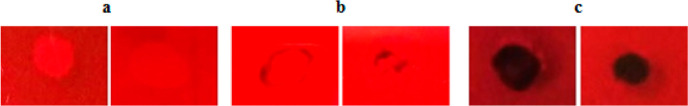
Classification of biofilms-producing *S. epidermidis* isolates using the CRA method. (a) Non-biofilm producers, (b) weak biofilm producers, and (c) strong biofilm producers.

After Stepanović’s classification of the isolates, the OD_630nm_ of the produced biofilms in the two different media were compared using the unpaired *T* test with Welch’s correction using GraphPad Prism software version 7.0. In TSB medium, 25 isolates showed significantly better biofilms (*p* < 0.05), whereas only eight isolates formed significantly better biofilms in M9 medium (*p* < 0.05). The differences between biofilms formed by the remaining 17 isolates were non-significant (*p* > 0.05). The CRA technique classified five (10%) isolates as strong biofilm producers, 26 (52%) as weak biofilm formers, and 19 (38%) isolates as non-biofilm formers on agar plates (Figure 2).

### 3.2 Genotypic Detection of Biofilm-Forming Genes

**Figure 3:**
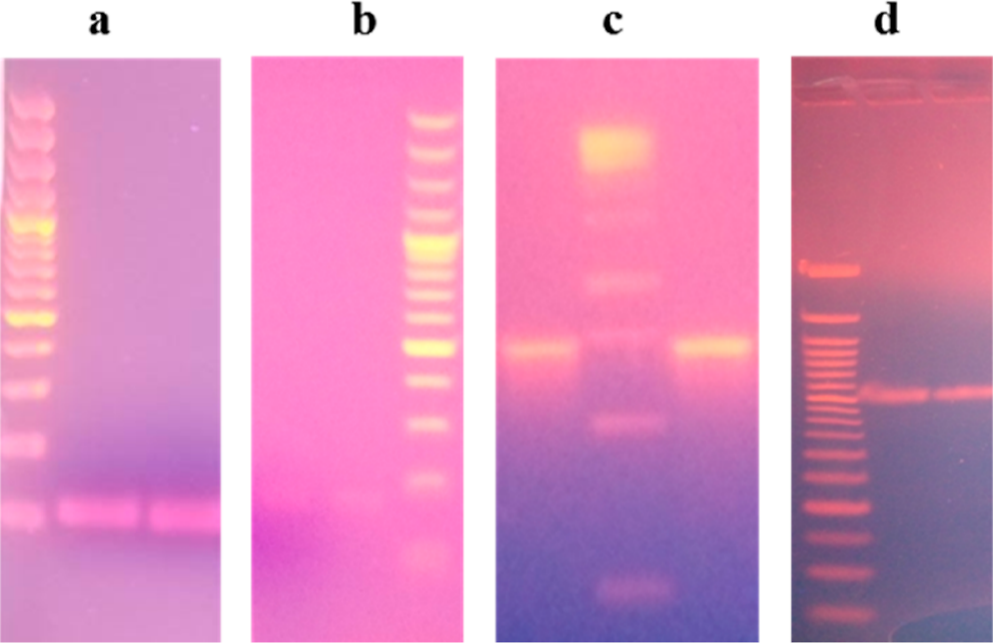
PCR amplifications of the targeted genes. (a) Amplified product of the *gseA* gene segment (124 bp) (b) *fruA* gene segment (179 bp), (c) *icaA* gene segment (295 bp), and (d) *icaB* gene segment (526 bp).

The performed PCR detected different biofilm-forming genes among the 50 *S. epidermidis* isolates. Among the 10 targeted genes, *fruA* was the most prevalent, being detected in 10 (20%) isolates, followed by *icaA* 9 (18%) and *icaB* 3 (6%) genes. The remaining seven genes were not detected in any of the isolates (Figure 3).

### 3.3 Antibiofilm Assay

In TSB medium, when the antibiofilm activity of CAR was tested against three strong biofilm-producing isolates (MU-5, MU-6, and MU-45), all the tested concentrations of CAR significantly reduced biofilm formation (*p* < 0.0001) as compared to the solvent control, while the differences among the three concentrations were found to be non-significant. The other two tested antibiofilm compounds (2-AB and 3-IA) significantly reduced biofilm formation (*p* < 0.0001 and *p* = 0.006, respectively) in TSB medium when used at the highest concentrations, that is, 200 μg/mL and 1 mg/mL, respectively; however, the other two tested concentrations did not reduce biofilm formation significantly (*p* > 0.05). Similarly, while testing the antibiofilm compounds in M9 medium against three strong biofilm-producing isolates (MU-8, MU-18, and MU-45), the highest concentrations of 2-AB and 3-IA significantly reduced biofilm formation (*p* = 0.0107 and *p* < 0.0001, respectively) as compared to the solvent control, whereas the reduction with other two concentrations was found to be non-significant (*p* > 0.05). Although the 3 mM concentration of CAR reduced biofilm formation in M9 medium, this reduction was statistically non-significant (*p* = 0.0713).

## 4 Discussion

Biofilm formation has been reported in different species of *Staphylococcus,* especially *S. aureus* and *S. epidermidis*, which is related to the contamination of biomedical devices. Different investigations have correlated the adherence of these microorganisms on various gadgets with the disease pathogenesis.^29,30^ For designing an effective antimicrobial therapy against *S. aureus* and *S. epidermidis* infection, the biofilm formation potential of the prevailing isolates needs to be investigated. Different phenotypic and genotypic methods have been used for the detection of biofilm-associated infections.^31^ We have used both phenotypic and genotypic approaches to detect the *in vitro* biofilms formed by clinical isolates of *S. epidermidis*.

In addition to PIA, the main adhesin of *S. epidermidis*, the extracellular matrix of staphylococcal biofilms contained amyloid fibrils, extracellular DNA, and other proteins. Different studies have reported the association of the *ica* operon and other genes with the biofilm formation ability of various *Staphylococcus* isolates. The *icaA* gene encodes *N*-acetylglucosaminyl transferase, which produces PIA oligomers, whereas the optimal efficiency of IcaA is supported by the product of the *icaD* gene. The externalization of the nascent polysaccharide of *Staphylococcus* is supported by the *icaC* gene product, whereas deacetylation of PIA is achieved by *N*-deacetylase, a product of *icaB*. Additionally, certain environmental conditions also affect the expression of the *ica* locus.^32^ Previous studies have reported a varying degree of the *ica* operon prevalence ranging from 27% in nasopharyngeal *S. epidermidis* isolates,^33^ to 45% in clinical isolates.^34^ In the current study, the *icaA* gene was detected in nine (18%) isolates, out of which three isolates also contained the *icaB* gene. The difference in prevalence is due to multiple factors including geographical variation, quorum sensing (negatively correlated with biofilm formation) and other alternative (*ica*-independent) mechanisms of biofilm formation by *S. epidermidis* isolates.^32^ Instead of mere gene presence, other factors including expression of genes under suitable conditions, neighboring microbial species including *Candida*,^35^ and nutritional and community based factors play a vital role in biofilm formation^36^ as no association was found between the presence of genes and phenotypic detection of biofilms formed by *S. epidermidis* isolates in our study. Similarly, other studies reported the presence of biofilm-associated genes in both biofilm-forming and non-biofilm-forming isolates; however, the expression of these genes was found to be significantly high among biofilm-forming isolates.^37^

The phenotypic detection of biofilm formation by the TCP method^24^ detected two isolates forming strong biofilms in each of the two tested media, where one isolate showed a strong biofilm in both media. This percentage (10%) of strong biofilm-producing *S. epidermidis* is comparable with that in other studies. Although another study has detected 16.6% of *S. epidermidis* isolates as moderate biofilm producers, no isolate could be categorized as a strong biofilm former.^34^ Chemically defined media (CDM), having additional amino acids (including l-lysine), glucose, purines, vitamins, and salts, demonstrated strong biofilm formation by 32.5% *S. epidermidis* isolates.^38^ The comparative OD_630nm_ analysis found the glucose supplemented TSB to be more suitable to *S. epidermidis* for biofilm formation because it supported significantly better biofilms (*p* < 0.05) in 25 (50%) isolates. A similar enhancement in biofilm formation by *S. epidermidis* due to glucose and other nutrient supplementation has also been reported earlier using CDM^38^ and TSB^1^ growth media. Although the CRA method also detected five (10%) isolates as strong biofilm producers, these isolates were different from the strong biofilm producers detected by the TCP and genotypic methods. Moreover, the CRA method was not able to differentiate between weak and moderate biofilm producers^39^ and was also reported to have limited reproducibility.^31^ Thus, despite being a simple and quick method, the CRA method has compromised applicability in laboratory settings as compared to the reliable TCP method.

Different antibiofilm compounds have been tested against multiple clinical pathogens including *Staphylococcus* species. This study evaluated the antibiofilm potential of previously reported (CAR and 2-AB) and novel 3-IA compounds against strong biofilm-producing *S. epidermidis* isolates. The use of toluene as an organic solvent for 2-AB and 3-IA was also evaluated. 3-IA has been reported for its antibiofilm activity against *Escherichia coli* and reducing the virulence of *Pseudomonas aeruginosa*;^40^ however, its antibiofilm activity has not been reported against *S. epidermidis*. We hereby report the significant antibiofilm activity of 3-IA (1 mg/mL) against *S. epidermidis* biofilms. Different studies have reported the antimicrobial as well as antibiofilm activity of CAR against different bacterial infections.^41,42^ Although CAR reduced biofilm formation significantly in supplemented TSB medium, it reduced biofilm formation in M9 medium only at lower concentrations, similar to the previously findings.^26^ The hydroxyl group of CAR has been reported for its activity against various bacterial pathogens.^43^ Out of the three tested concentrations of 2-AB, significant reduction in *S. epidermidis* biofilms was found with 200 μg/mL, similar to the previously reported concentration-dependent reduction in biofilm formation by *S. aureus* and *Candida albicans* isolates.^27^ Clinical *S. epidermidis* isolates demonstrated adequate *in vitro* biofilm formation in different media, as detected by phenotypic and genotypic methods. The rapidly increasing biofilm-forming ability of *S. epidermidis* and antibiofilm potential of the tested compounds highlighted its importance while devising the strategies of treatment and control of *S. epidermidis* infections in clinical settings.

## References

[ref1] Mathur T.; Singhal S.; Khan S.; Upadhyay D.; Fatma T.; Rattan A. Detection of biofilm formation among the clinical isolates of staphylococci: an evaluation of three different screening methods. Indian J. Med. Microbiol. 2006, 24, 25–29. 10.4103/0255-0857.19890. 16505551

[ref2] Sakimura T.; Kajiyama S.; Adachi S.; Chiba K.; Yonekura A.; Tomita M.; Koseki H.; Miyamoto T.; Tsurumoto T.; Osaki M. Biofilm-forming *Staphylococcus epidermidis* expressing vancomycin resistance early after adhesion to a metal surface. BioMed Res. Int. 2015, 2015, 943056 10.1155/2015/943056. 25802873 PMC4329865

[ref3] Lade H.; Park J. H.; Chung S. H.; Kim I. H.; Kim J.-M.; Joo H.-S.; Kim J.-S. Biofilm formation by *Staphylococcus aureus* clinical isolates is differentially affected by glucose and sodium chloride supplemented culture media. J. Clin. Med. 2019, 8, 1853 10.3390/jcm8111853. 31684101 PMC6912320

[ref4] Kloos W. E.; Bannerman T. L. Update on clinical significance of coagulase-negative staphylococci. Clin. Microbiol. Rev. 1994, 7, 117–140. 10.1128/cmr.7.1.117. 8118787 PMC358308

[ref5] O’Gara J. P.; Humphreys H. *Staphylococcus epidermidis biofilms*: importance and implications. J. Med. Microbiol. 2001, 50, 582–587. 10.1099/0022-1317-50-7-582. 11444767

[ref6] Ammendolia M.; Di Rosa R.; Montanaro L.; Arciola C.; Baldassarri L. Slime production and expression of the slime-associated antigen by staphylococcal clinical isolates. J. Clin. Microbiol. 1999, 37, 3235–3238. 10.1128/jcm.37.10.3235-3238.1999. 10488184 PMC85536

[ref7] Arciola C. R.; Baldassarri L.; Montanaro L. Presence of *icaA* and *icaD* genes and slime production in a collection of staphylococcal strains from catheter-associated infections. J. Clin. Microbiol. 2001, 39, 2151–2156. 10.1128/jcm.39.6.2151-2156.2001. 11376050 PMC88104

[ref8] Cramton S. E.; Gerke C.; Schnell N. F.; Nichols W. W.; Götz F. The intercellular adhesion (ica) locus is present in *Staphylococcus aureus* and is required for biofilm formation. Infect. Immun. 1999, 67, 5427–5433. 10.1128/iai.67.10.5427-5433.1999. 10496925 PMC96900

[ref9] Speziale P.; Pietrocola G.; Foster T. J.; Geoghegan J. A. Protein-based biofilm matrices in Staphylococci. Front. Cell. Infect. Microbiol. 2014, 4, 171 10.3389/fcimb.2014.00171. 25540773 PMC4261907

[ref10] Halim R. M. A.; Kassem N. N.; Mahmoud B. S. Detection of biofilm producing staphylococci among different clinical isolates and its relation to methicillin susceptibility. Open Access Maced. J. Med. Sci. 2018, 6, 1335 10.3889/oamjms.2018.246. 30159052 PMC6108796

[ref11] Christensen G. D.; Simpson W. A.; Younger J.; Baddour L.; Barrett F.; Melton D.; Beachey E. Adherence of coagulase-negative staphylococci to plastic tissue culture plates: a quantitative model for the adherence of staphylococci to medical devices. J. Clin. Microbiol. 1985, 22, 996–1006. 10.1128/jcm.22.6.996-1006.1985. 3905855 PMC271866

[ref12] Kaiser T. D. L.; Pereira E. M.; dos Santos K. R. N.; Maciel E. L. N.; Schuenck R. P.; Nunes A. P. F. Modification of the Congo red agar method to detect biofilm production by *Staphylococcus epidermidis*. Diagn. Microbiol. Infect. Dis. 2013, 75, 235–239. 10.1016/j.diagmicrobio.2012.11.014. 23313084

[ref13] Kord M.; Ardebili A.; Jamalan M.; Jahanbakhsh R.; Behnampour N.; Ghaemi E. A. Evaluation of biofilm formation and presence of ica genes in *Staphylococcus epidermidis* clinical isolates. Osong Public Health Res. Perspect. 2018, 9, 160 10.24171/j.phrp.2018.9.4.04. 30159221 PMC6110329

[ref14] Schiebel J.; Noack J.; Rödiger S.; Kammel A.; Menzel F.; Schwibbert K.; Weise M.; Weiss R.; Böhm A.; Nitschke J. Analysis of three-dimensional biofilms on different material surfaces. Biomater. Sci. 2020, 8, 3500–3510. 10.1039/d0bm00455c. 32432585

[ref15] Chen W.; Xie T.-T.; Zeng H. Formation, antibiotic resistance, and control strategies of *Staphylococcus epidermidis* biofilm. Bacterial Biofilms; IntechOpen, 2019.

[ref16] Abidi S. H.; Ahmed K.; Kazmi S. U. The antibiofilm activity of Acetylsalicylic acid, Mefenamic acid, acetaminophen against biofilms formed by *P. aeruginosa* and *S. epidermidis*. J. Pak. Med. Assoc. 2019, 69, 1493–1495. 10.5455/jpma.295488. 31622303

[ref17] Mohsen A.; Gomaa A.; Khalaf A.; Kamal M.; Mokhtar S.; Mohamed H.; Salah I.; Abbas R.; Ali S.; Abd El-Baky R. M. Antibacterial, anti-biofilm activity of some non-steroidal anti-inflammatory drugs and N-acetyl cysteine against some biofilm producing uropathogens. Am. J. Epidemiol. Infect. Dis. 2015, 3, 1–9. 10.12691/ajeid-3-1-1.

[ref18] Alem M. A.; Douglas L. J. Effects of aspirin and other nonsteroidal anti-inflammatory drugs on biofilms and planktonic cells of *Candida albicans*. Antimicrob. Agents Chemother. 2004, 48, 41–47. 10.1128/aac.48.1.41-47.2004. 14693516 PMC310207

[ref19] Abidi S. H.; Sherwani S. K.; Siddiqui T. R.; Bashir A.; Kazmi S. U. Drug resistance profile and biofilm forming potential of Pseudomonas aeruginosa isolated from contact lenses in Karachi-Pakistan. BMC Ophthalmol. 2013, 13, 57 10.1186/1471-2415-13-57. 24134792 PMC3852958

[ref20] Taj Y.; Essa F.; Aziz F.; Kazmi S. U. Study on biofilm-forming properties of clinical isolates of *Staphylococcus aureus*. J. Infect. Dev. Countries 2012, 6, 403–409. 10.3855/jidc.1743. 22610706

[ref21] Ikeda Y.; Ohara-Nemoto Y.; Kimura S.; Ishibashi K.; Kikuchi K. PCR-based identification of *Staphylococcus epidermidis* targeting gseA encoding the glutamic-acid-specific protease. Can. J. Microbiol. 2004, 50, 493–498. 10.1139/w04-055. 15381974

[ref22] Hassan A.; Usman J.; Kaleem F.; Omair M.; Khalid A.; Iqbal M. Evaluation of different detection methods of biofilm formation in the clinical isolates. Braz. J. Infect. Dis. 2011, 15, 305–311. 10.1590/s1413-86702011000400002. 21860999

[ref23] Kerk S. K.; Lai H. Y.; Sze S. K.; Ng K. W.; Schmidtchen A.; Adav S. S. Bacteria display differential growth and adhesion characteristics on human hair shafts. Front. Microbiol. 2018, 9, 2145 10.3389/fmicb.2018.02145. 30245682 PMC6137140

[ref24] Stepanović S.; Vuković D.; Hola V.; Di Bonaventura G. D.; Djukić S.; Cirković I.; Ruzicka F. Quantification of biofilm in microtiter plates: overview of testing conditions and practical recommendations for assessment of biofilm production by staphylococci. APMIS 2007, 115, 891–899. 10.1111/j.1600-0463.2007.apm_630.x. 17696944

[ref25] Freeman D.; Falkiner F.; Keane C. New method for detecting slime production by coagulase negative staphylococci. J. Clin. Pathol. 1989, 42, 872–874. 10.1136/jcp.42.8.872. 2475530 PMC1142068

[ref26] Burt S. A.; Ojo-Fakunle V. T.; Woertman J.; Veldhuizen E. J. The natural antimicrobial carvacrol inhibits quorum sensing in Chromobacterium violaceum and reduces bacterial biofilm formation at sub-lethal concentrations. PLoS One 2014, 9, e93414 10.1371/journal.pone.0093414. 24691035 PMC3972150

[ref27] Tan Y.; Leonhard M.; Moser D.; Ma S.; Schneider-Stickler B. Antibiofilm efficacy of curcumin in combination with 2-aminobenzimidazole against single-and mixed-species biofilms of *Candida albicans* and *Staphylococcus aureus*. Colloids Surf., B 2019, 174, 28–34. 10.1016/j.colsurfb.2018.10.079. 30412864

[ref28] Amer M. A.; Wasfi R.; Attia A. S.; Ramadan M. A. Indole derivatives obtained from Egyptian *Enterobacter* sp. soil isolates exhibit antivirulence activities against uropathogenic *Proteus mirabilis*. Antibiotics 2021, 10, 363 10.3390/antibiotics10040363. 33805493 PMC8065651

[ref29] Atshan S. S.; Nor Shamsudin M.; Sekawi Z.; Lung L. T. T.; Hamat R. A.; Karunanidhi A.; Mateg Ali A.; Ghaznavi-Rad E.; Ghasemzadeh-Moghaddam H.; Chong Seng J. S.; et al. Prevalence of adhesion and regulation of biofilm-related genes in different clones of *Staphylococcus aureus*. J. Biomed. Biotechnol. 2012, 2012, 1–10. 10.1155/2012/976972. 22701309 PMC3372070

[ref30] Götz F. *Staphylococcus* and biofilms. Mol. Microbiol. 2002, 43, 1367–1378. 10.1046/j.1365-2958.2002.02827.x. 11952892

[ref31] Manandhar S.; Singh A.; Varma A.; Pandey S.; Shrivastava N. Evaluation of methods to detect in vitro biofilm formation by staphylococcal clinical isolates. BMC Res. Notes 2018, 11, 714 10.1186/s13104-018-3820-9. 30305150 PMC6180658

[ref32] Arciola C. R.; Campoccia D.; Ravaioli S.; Montanaro L. Polysaccharide intercellular adhesin in biofilm: structural and regulatory aspects. Front. Cell. Infect. Microbiol. 2015, 5, 7 10.3389/fcimb.2015.00007. 25713785 PMC4322838

[ref33] Los R.; Sawicki R.; Juda M.; Stankevic M.; Rybojad P.; Sawicki M.; Malm A.; Ginalska G. A comparative analysis of phenotypic and genotypic methods for the determination of the biofilm-forming abilities of *Staphylococcus epidermidis*. FEMS Microbiol. Lett. 2010, 310, 97–103. 10.1111/j.1574-6968.2010.02050.x. 20722741

[ref34] Nasr R. A.; AbuShady H. M.; Hussein H. S. Biofilm formation and presence of *icaAD* gene in clinical isolates of staphylococci. Egypt. J. Med. Hum. Genet. 2012, 13, 269–274. 10.1016/j.ejmhg.2012.04.007.

[ref35] Phuengmaung P.; Panpetch W.; Singkham-In U.; Chatsuwan T.; Chirathaworn C.; Leelahavanichkul A. Presence of *Candida tropicalis* on *Staphylococcus epidermidis* biofilms facilitated biofilm production and *Candida d*issemination: An impact of fungi on bacterial biofilms. Front. Cell. Infect. Microbiol. 2021, 11, 763239 10.3389/fcimb.2021.763239. 34746032 PMC8569676

[ref36] Jefferson K. K. What drives bacteria to produce a biofilm?. FEMS Microbiol. Lett. 2004, 236, 163–173. 10.1111/j.1574-6968.2004.tb09643.x. 15251193

[ref37] Amin M.; Navidifar T.; Saleh Shooshtari F. S.; Rashno M.; Savari M.; Jahangirmehr F.; Arshadi M. Association between biofilm formation, structure, and the expression levels of genes related to biofilm formation and biofilm-specific resistance of *Acinetobacter baumannii* strains isolated from burn infection in Ahvaz, Iran. Infect. Drug Resist. 2019, 12, 3867 10.2147/idr.s228981. 31853190 PMC6914661

[ref38] Cafiso V.; Bertuccio T.; Santagati M.; Campanile F.; Amicosante G.; Perilli M.; Selan L.; Artini M.; Nicoletti G.; Stefani S. Presence of the *ica* operon in clinical isolates of *Staphylococcus epidermidis* and its role in biofilm production. Clin. Microbiol. Infect. 2004, 10, 1081–1088. 10.1111/j.1469-0691.2004.01024.x. 15606635

[ref39] Arciola C. R.; Campoccia D.; Gamberini S.; Cervellati M.; Donati E.; Montanaro L. Detection of slime production by means of an optimised Congo red agar plate test based on a colourimetric scale in *Staphylococcus epidermidis* clinical isolates genotyped for *ica* locus. Biomaterials 2002, 23, 4233–4239. 10.1016/s0142-9612(02)00171-0. 12194526

[ref40] Lee J. H.; Cho M. H.; Lee J. 3-Indolylacetonitrile decreases *Escherichia coli* O157: H7 biofilm formation and *Pseudomonas aeruginosa* virulence. Environ. Microbiol. 2011, 13, 62–73. 10.1111/j.1462-2920.2010.02308.x. 20649646

[ref41] Baygar T.; Ugur A.; Sarac N.; Balci U.; Ergun G. Functional denture soft liner with antimicrobial and antibiofilm properties. J. Dent. Sci. 2018, 13, 213–219. 10.1016/j.jds.2017.10.002. 30895123 PMC6388823

[ref42] Nostro A.; Cellini L.; Zimbalatti V.; Blanco A. R.; Marino A.; Pizzimenti F.; Giulio M. D.; Bisignano G. Enhanced activity of carvacrol against biofilm of *Staphylococcus aureus* and *Staphylococcus epidermidis* in an acidic environment. APMIS 2012, 120, 967–973. 10.1111/j.1600-0463.2012.02928.x. 23030501

[ref43] Ultee A.; Bennik M.; Moezelaar R. The phenolic hydroxyl group of carvacrol is essential for action against the food-borne pathogen *Bacillus cereus*. Appl. Environ. Microbiol. 2002, 68, 1561–1568. 10.1128/aem.68.4.1561-1568.2002. 11916669 PMC123826

